# Meteorological factors affecting dengue incidence in Davao, Philippines

**DOI:** 10.1186/s12889-018-5532-4

**Published:** 2018-05-15

**Authors:** Jesavel A. Iguchi, Xerxes T. Seposo, Yasushi Honda

**Affiliations:** 1 Department of Health Care Policy and Health Economics, Graduate School of Comprehensive Human Sciences, Ibaraki, 305-8577 Japan; 2 Department of Environmental Engineering, Graduate School of Engineering, Kyoto University, Kyoto, 615-8530 Japan; 3 Faculty of Health and Sports Sciences, University of Tsukuba, Ibaraki, 305-8577 Japan

**Keywords:** Dengue, Meteorological, *Aedes*, Rainfall, Temperature, Distributed lag nonlinear model, Wavelet analysis, Time series, Philippines

## Abstract

**Background:**

Dengue fever is a major public health concern in the Philippines, and has been a significant cause of hospitalizations and deaths among young children. Previous literature links climate change to dengue, and with increasingly unpredictable changing climate patterns, there is a need to understand how these meteorological variables affect dengue incidence in a highly endemic area.

**Methods:**

Weekly dengue incidences (2011–2015) in Davao Region, Philippines were obtained from the Department of Health. Same period of weekly local meteorological variables were obtained from the National Climatic Data Center (NCDC) and the National Oceanic and Atmospheric Administration (NOAA). Wavelet coherence analysis was used to determine the presence of non-stationary relationships, while a quasi-Poisson regression combined with distributed lag nonlinear model (DLNM) was used to analyze the association between meteorological variables and dengue incidences.

**Results:**

Significant periodicity was detected in the 7 to 14-week band between the year 2011–2012 and a 26-week periodicity from the year 2013–2014. Overall cumulative risks were particularly high for rainfall at 32 mm (RR: 1.67, 95% CI: 1.07–2.62), while risks were observed to increase with increasing dew point. On the other hand, lower average temperature of 26 °C has resulted to an increased RR of dengue (RR: 1.96, 95% CI: 0.47–8.15) while higher temperature from 27 °C to 31 °C has lower RR.

**Conclusions:**

The observed possible threshold levels of these meteorological variables can be integrated into an early warning system to enhance dengue prediction for better vector control and management in the future.

**Electronic supplementary material:**

The online version of this article (10.1186/s12889-018-5532-4) contains supplementary material, which is available to authorized users.

## Background

Dengue fever is a common mosquito-borne viral disease of humans transmitted by the bite of an infected female adult mosquito namely the *Aedes aegypti* as the primary vector and *Aedes albopictus* as the secondary vector. *Aedes aegypti* is a holometabolous insect with a life cycle consisting of four stages, namely: egg, instars, pupa and adult [1]. It is characterized with a round or globular head structure, a white flat scales in the middle vertex and a slender, black, long and cylindrical proboscis [2]. Dengue can cause fatal complications such as dengue haemorrhagic fever (DHF) and dengue shock syndrome [3] . Dengue virus belongs to the single-stranded RNA virus of the Flaviviridae family that has four viral serotypes namely; DEN-1, DEN-2, DEN-3, DEN-4 [4]. The adult female *Aedes aegypti,* the primary vector is a small, black mosquito with white markings around its body. Frequently, mosquito vectors lay its eggs in places within or near human dwellings; with only female adult mosquitoes transmitting the dengue virus [3, 5].

Dengue remains a major public health concern in tropical and subtropical areas [3]. Over the last 50 years, dengue incidence has increased by 30-fold and around 2.5 billion people live in areas where dengue is endemic. Moreover, an estimated 50–200 million cases of dengue infections occur annually in the world [6]. The spread of dengue may be partly due to the increase of international travel, unplanned urbanization, rapid increase in population growth, lack of effective vector management, climate change and extreme weather events, and poor socio-economic status [7–9].

Dengue disease has risen in an alarming state in the Philippines in recent years. From January 1st to August 6th of 2016, the Philippines’ Department of Health (DOH) reported an estimated suspected dengue cases of 84,085 in the country, which is 15.8% higher compared to the same period of last year in 2015 with only 72,627 reported cases; out of this, 372 resulted to death [10]. Out of the 10 Association of South East Asian Nations (ASEAN), the Philippines ranked fourth for having the highest number of dengue cases as of 2012 [11]. This alarming rate is partly due to several factors such as environmental degradation, climatic condition, lack of clean water supply, inappropriate waste disposal and management, rapid urbanization, increasing population, and poor mosquito surveillance and control system all contributed to the increasing number of dengue cases in the country [12].

The increasing dengue incidence worldwide is caused by several factors and one of them, which is our primary focus, are the meteorological factors. Change in these factors is believed to influence people’s health through the spread of vector-borne diseases [8]. For example, meteorological factors such as temperature, rainfall, and humidity influence the life stages of female adult *Aedes* mosquitoes. A warm temperature is important to adult mosquitos’ behavior and maturation, especially the larval development rate is shortened [13, 14]. In addition, rainfall provides plenty of breeding sites for mosquito vectors such as puddles, while humidity affects the adult mosquitoes’ survival and biting frequency [15].

Many countries have conducted studies on the relationship between meteorological factors and dengue cases. For instance, in the temperature and dengue relationship studies, different lagged effect was observed. An increase in RR of dengue was reported to be related with an increase in minimum and maximum temperature by up to 1 to 2-month lag period in Brazil [16] and a lag of 1 month for maximum temperature in Mexico [17]. Meanwhile, a longer lag of up to 3 to 4 months was observed in Australia [18] and Barbados [19]. Furthermore, cumulative rainfall and dengue have also been found to have a varying lag effect; such as a 2-week lag in Mexico [20], a 4-week lag in Thailand [21], and a 10-week lag in Taiwan [22]. The differences on the effects of weather on dengue incidences could be due to the different variations of the amount of rainfall or the range of temperatures in the different regions with respect to their geographical locations [23].

Various approaches have been utilized to estimate the risks and the associated delayed risks of various local meteorological variables on dengue incidence, with a variety of linear [24] and non-linear models [25]. Recent methodological advancements have resulted to the utilization of a distributed lag nonlinear model (DLNM) [26], taking into account the bi-dimensionality of the risks, exposure and lag components, evident in previous studies [27–30]. Further methodological specifications of DLNM are extensively discussed elsewhere [26, 31, 32]. Here, we aim to elucidate the effects of the local meteorological variables, one of the significant driving forces on dengue transmission [33]. This will enable us to determine the period of high risk of dengue infection since we hypothesized that, local meteorological factors may have an impact on the dengue incidences in Davao Region, Philippines. Clarifying the effects of these meteorological factors on dengue could provide an insight into the seasonal mechanisms of the disease thereby rendering information to understand the complex relationship between meteorological factors and health. Moreover, this study could provide valuable information to health officers for a more effective management of the disease for its control and prevention.

## Methods

### Study area

**Fig. 1:**
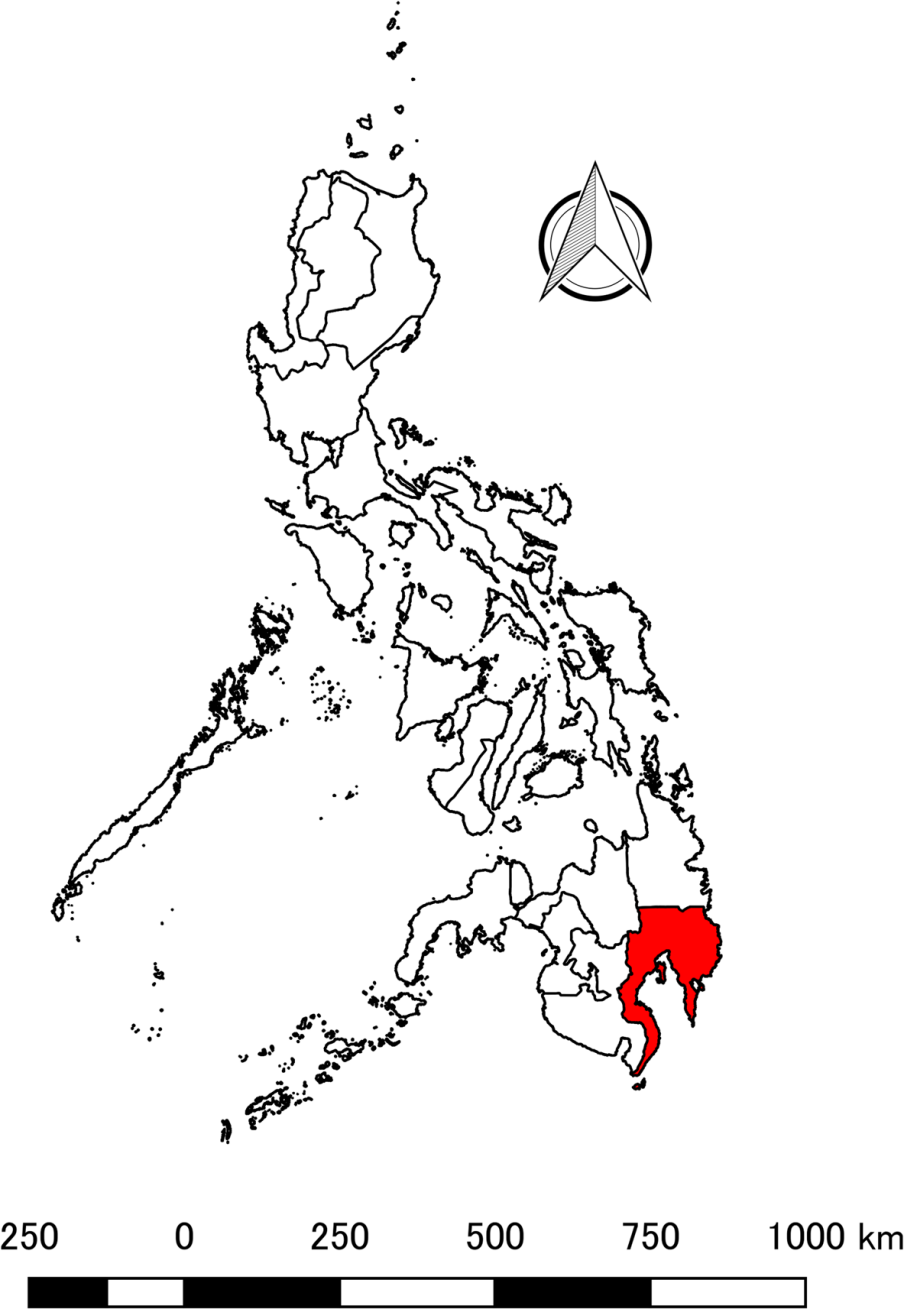
Geographical location of Davao Region, Philippines. Red-colored area indicates the location of Davao Region, which situated in the Southernmost Island of Mindanao, Philippines

The study site is located in Davao Region, which is situated at the southeastern portion of the third largest island in the Philippines called Mindanao at 7°05′ latitude North and 125°35′ longitude East (Fig. 1). It is comprised of five provinces namely Compostela Valley, Davao del Norte, Davao del Sur, Davao Oriental, Davao Occidental [34]. It has a total land area of 20,357 km^2^ and with a total population of 4,893,318 in 2015 [35], making it the country’s fifth fastest growing in terms of the population and 5% in the whole country [36]. The climate is tropical with two distinct seasons: a dry and wet season. According to the Philippine Atmospheric Geophysical and Astronomical Services Administration (PAGASA), wet season occurs from the months of April to October, while dry season occurs from the months of November to March [37]. There are four existing climate types in the Philippines based on the country’s modified Coronas classification, namely Type I (dry from November to April, wet from May to October), Type II (seasonal rainfall from November to December), Type III (same as Type I, but with maximum rainfall from May to October) and Type IV (even distribution of rainfall year-round) [38]. Davao region is classified under Type IV with an annual average temperature of 28 °C; a temperature average shared with other major cities across the country [39].

### Data collection

We conducted a retrospective time-series study of weekly dengue incidences and meteorological variables in Davao Region from 2011 to 2015. Weekly secondary data of dengue incidences were obtained from the Department of Health-Davao Region (DOH-Region XI) [40] and was freely available through formal data request. The case classification for the dengue incidences used in this study were based on the standard definitions of DOH and were not laboratory confirmed. The meteorological variables of interest, daily average temperature (°C), cumulative rainfall per week (mm), and dew point (°C) reported by Francisco Bangoy International Airport in Davao City, Philippines, were extracted from the climate prediction center of the US National Weather Service website NOAA and NCDC (https://www.ncdc.noaa.gov/), which were then aggregated to weekly measures. Daily average temperature was computed as the mean of daily maximum and minimum temperatures. Weekly average temperature, dew point, and weekly cumulative rainfall were generated from the average of the seven-day daily observations for each meteorological variable.

### Statistical analyses

#### Wavelet coherence analysis

This was performed to detect changes in the periodicity in the dengue incidence time series. According to Cazelles et al. [41] wavelet approach extracts the time and frequency of a time series and it is the most efficient method in studying non-stationary data. It transforms a time series into a wave [42]. Furthermore, wavelet was used in this study to determine whether the presence of a particular periodic frequency at a given time in dengue incidences corresponded to the presence of the same periodic frequency at the same time in the given meteorological variables [43].

#### Distributed lag nonlinear model

A quasi-Poisson time series model coupled with DLNM, which was introduced by Armstrong in 2006 [44] and expanded by Gasparrini in 2010 [31, 32], was used to assess the impact of meteorological variables on dengue incidences. The relationship between meteorological factors and diseases are seldom linear, and the former’s effect are commonly delayed [45]. DLNM uses a cross-basis function, which enables users to explore the association between the exposure variable and its lag, which is in weeks in this instance [46]. We assumed that the reported dengue incidences, *y,* at week *t,* follows an over-dispersed quasi-Poisson distribution and can be written as shown below:

#### y_t_~quasipoisson

1 $$ \log \left(\mathrm{E}\left({y}_t\right)\right]=\upalpha +{\beta}_1{Rainfall}_{t,l}+ ns\left( Week,4\times 5\right) $$

Where *y*_*t*_ refers to the weekly dengue incidences on week *t*, while *t* denotes the week of the observation, *α* is the model intercept, *Rainfall*_*t,l*_ is the cross-basis matrix for cumulative rainfall as a covariate on time (*t*) with 4 degrees of freedom (*df*), *l* is the lag in weeks, *ns* is the smoothing parameter specified on a natural cubic spline (NCS), *Week* is assigned to control for the seasonal variation.

Moreover, we conducted the DLNM analysis for a maximum lag of 12 weeks. For other meteorological variables, we replaced the cross-basis of the rainfall in Eq. 1 with the respective cross-basis of the other meteorological variables. Model selection was facilitated using three performance indicators namely, Quasi-Akaike Information Criterion (QAIC), Root Mean Squared Error (RMSE) and R-squared as indicated in Table 1. For comparison, we also assumed a simple linear regression with the same specifications.

**Table 1:** The QAIC, RMSE and pseudo R-squared values for the selected models

Models		QAIC	RMSE	pseudo R-squared
Intercept only		13,869.39	–	–
Simple Linear Models				
	w/ Rainfall	6088.93	0.3292689	0.6785798
	w/ Average temperature	5866.015	0.3222838	0.6954379
	w/ Dew point	5456.907	0.3183454	0.7251268^a^
DLNM Models				
	w/ Rainfall	4313.961	0.241543	0.8085139
	w/ Average temperature	4291.979	0.251437	0.8112628
	w/ Dew point	4002.741	0.2350322	0.8321903^b^

*QAIC* Quasi-Akaike Information Criterion, *RMSE* Root Mean Squared Error

^a^Best predictor among simple linear models

^b^Best predictor among the DLNM models

All the statistical analyses were performed using R software program, version 3.3.1 [47], while the map figure was created using QGIS version 3.0 [48].

#### Sensitivity analyses

The passive surveillance system, utilized by the country, is prone to under-reporting and may have considerable impact on the effects estimate [49]. To establish the robustness of the effects estimates, we applied an expansion factor (EF) of 7.0 for the suspected number of cases [11, 50]. EFs have been utilized to adjust for the underreporting of the total number of cases from surveillance systems [50]. For model performance, we further analyzed for a multivariate model with DLNM and simple linear models (in Additional file 1: Table S1).

## Results

### Dengue incidence distribution

**Fig. 2:**
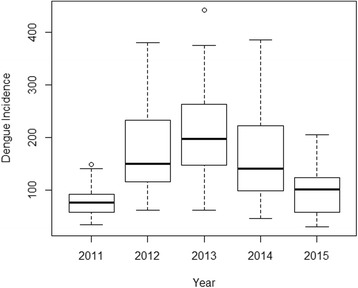
Annual distribution of dengue incidences in Davao Region, 2011–2015. Dengue incidences increased from 4115 cases in 2011 to 9507 cases in 2012. The highest number of dengue incidences among the study period was recorded around the year 2013 with 10,762 cases. Dengue incidence gradually decreased in 2014 with 8643 cases towards 2015 with 5142 cases

Table 2 shows the descriptive statistics of both weekly dengue incidences and local meteorological variables in Davao Region from 2011 to 2015. From 2011 to 2015, there were 38,169 dengue cases reported at the DOH-Davao Region. In the study period, dengue increased from 4115 cases in 2011 to 9507 cases in 2012, and reached a peak in the year 2013 with 10,762 cases, the highest reported number of dengue cases within the study period. Then, it gradually decreased to 8643 in 2014 and at 5142 cases in 2015 (Fig. 2). The highest reported number of dengue was at the 6^th^ week of the year 2013 around the month of February (Additional files 2 and 3: Figures S2 and S3A) with 442 cases while the lowest was in the 13^th^ week around the month of March 2015 with 30 cases.

**Table 2:** Descriptive statistics of the weekly dengue incidences and local meteorological variables in Davao Region, 2011–2015

	Minimum	Maximum	Mean	Standard deviation
Dengue incidence	30	442	146.80	85.91
Average temperature (°C)	25.9	30.9	28.4	0.8
Rainfall (mm)	0.0	102.3	14.1	16.8
Dew point (°C)	21.3	25.9	24.5	0.8

**Fig. 3:**
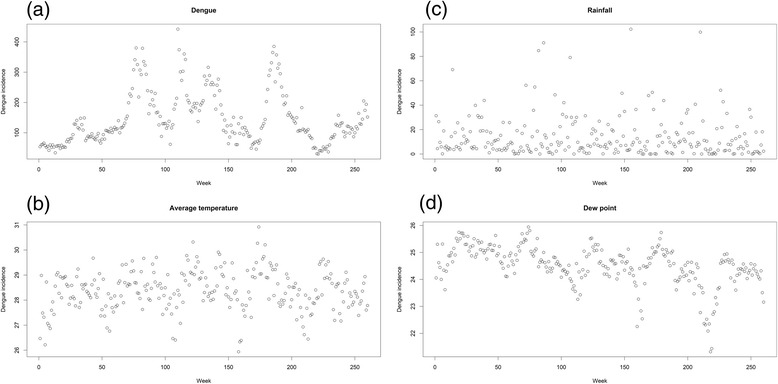
Time series plot of weekly dengue incidences and local meteorological variables in Davao Region from 2011 to 2015. (**a**) Dengue incidences, (**b**) Average temperature (°C), (**c**) Cumulative rainfall (mm) and (**d**) Dew point (°C)

Figure 3 represents the time trend of the weekly dengue incidences and local meteorological variables in Davao Region. The data showed that the number of dengue incidences were high from the year 2012, 2013 and 2014 (Fig. 3a). A higher number of dengue generally occurred from the rainy months of June throughout October (Additional file 3: Figure S3A), which consequently falls within the same period of the annual monsoon rains.

### Temperature and rainfall distribution

The highest weekly average temperature recorded within the study period was 30.9 °C in the 18^th^ week of 2014 around the month of May (Fig. 3b and Additional file 2: Figure S2), which is the second hottest month of the year after April, followed by 30.3 °C in the 18^th^ week of 2013 on the same month. Meanwhile, the lowest weekly average temperature recorded was 25.9 °C in the 2^nd^ week of 2014 around the month of January (Additional file 3: Figure S3B).

Between the study period from 2011 to 2015, 2012 has the largest volume of cumulative rainfall recorded at around 823.0 mm, while the smallest volume of rainfall was in 2015 with 604.0 mm (Table 3 and Additional file 4: Figure S1). The highest weekly cumulative rainfall reported was around the 51st week of 2013 around the month of December with 102.3 mm of rainfall (Fig. 3c and Additional file 3: Figure S3C).

**Table 3:** Annual distribution of dengue incidences and local meteorological variables in Davao Region, 2011–2015

Period	Dengue incidences	Average temperature (°C)	Rainfall (mm)	Dew point (°C)
2011	4115	28.2	744.5	25.1
2012	9507	28.4	823.0	24.8
2013	10,762	28.6	796.4	24.5
2014	8643	28.5	697.7	24.4
2015	5142	28.2	604.0	23.8

### Coherence between local meteorological variables and dengue

**Fig. 4:**
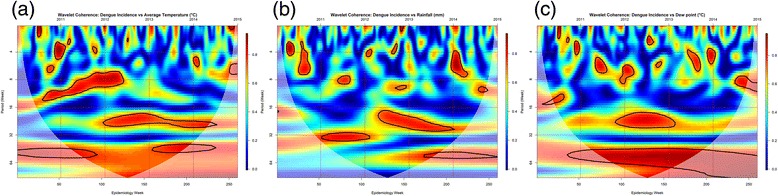
Wavelet coherence analysis between weekly dengue incidences and local meteorological variables in Davao Region from 2011 to 2015. Blue color represents low coherence, while red color shows high coherence. The black lines represent the areas where coherence is significantly high (95% confidence level). The cone of influence shown as a white curve indicates the region not influenced by edge effects. (**a**) Average temperature (°C), (**b**) Cumulative rainfall (mm), and (**c**) Dew point (°C)

Wavelet coherence analysis was used to detect periodicity between dengue incidences and local meteorological variables. In Fig. 4, the coherence between dengue incidences and local meteorological variables varied at different periods and the periodicity of the signals were different through time. For the average temperature and dengue incidences, there were significant periodicities detected in the 7- to 14-week band between the year 2011–2012 and a 26-week periodicity was also observed from the year 2013–2014. There were also several mild periodicities at 2- to 6-week periods that appear in an occasional pattern (Fig. 4a). Likewise, for the cumulative rainfall and dengue incidences, wavelet analysis showed a significant 32-week periodicity band from 2011 to 2012 and an 18- to 30-week periodicity from 2013 to 2014. Furthermore, several high periodicities were also observed in an infrequent pattern at around 2- to 10-week bands (Fig. 4b). For dew point and dengue incidences, wavelet results revealed a significant periodicity of a 20- to 26-week from 2012 to 2013 as well as a 50- to 60-week band from 2011 to 2015. There were also several high frequency periodicities at 2- to 6-week (Fig. 4c).

### Relationship of climate variables and dengue

**Fig. 5:**
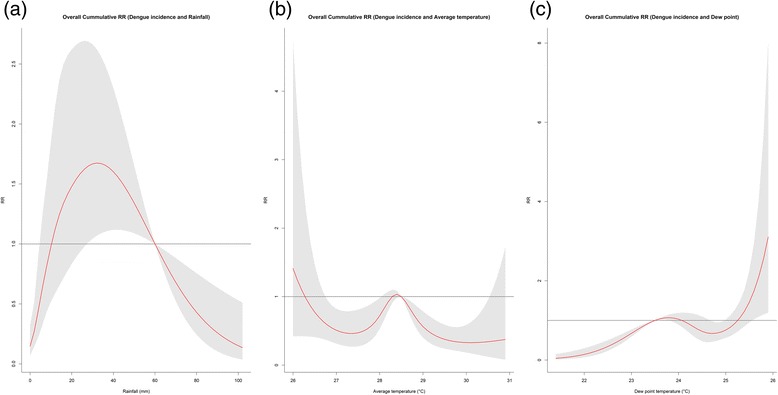
Estimated overall effects of the RR of dengue incidences relative to the meteorological variables. (**a**) Rainfall (mm), (**b**) Average temperature (°C) and (**c**) Dew point (°C) with a maximum lag of 12 weeks, using a “natural cubic B-spline-natural cubic spline” DLNM. The center red line represents the estimated spline curve, while the shadow shows 95% CI

To analyze the relationship between local meteorological variables and dengue incidences, a quasi-Poisson time series combined with DLNM was used. The effect of rainfall on dengue were different between low and high cumulative rainfall. As shown in the overall cumulative plot, risk was gradually increasing from 20 to 40 mm of rainfall, with a peak at 32 mm (RR: 1.67, 95% CI: 1.07–2.62). On the other hand, lower average temperature of 26 °C has resulted to an increased RR of dengue (RR: 1.96, 95% CI: 0.47–8.15) while higher temperature from 27 °C to 31 °C has lower RR (Fig. 5b). Meanwhile, for dew point, risks were increasing 25.3 °C. The highest RR was observed at dew point value of 26 °C (RR: 3.10, 95% CI: 1.20–8.06) (Fig. 5c).

## Discussion

In this study, we have observed significant but varying non-stationary periodicities between the local meteorological variables and that of dengue incidence. Further analysis indicated the effects of the varying levels of meteorological variables on dengue incidence. Findings from our study can be utilized for an integrated dengue early warning system, relevant for disease control and management.

In Fig. 4, we have observed significant, but varying inter-annual periodicities of dengue incidence with respect to the specific meteorological variables of interest. In particular, there are consistent longer significant bands from the 16th to 32nd week, for all the three local variables, which coincides with the summer month of April and gradually transitions towards the rainy month of August. There are also small pockets of significant mild periodicities in the 4th to 16th week across the years amongst the three meteorological variables, however, we have observed a significant inter-annual periodicity (2010–2012) from the 6th week to the 15th week, dry season, apparent only with average temperature. Though some studies have noted that mosquito activity would be high in the rainy periods, there are indications that even in dry season mosquito activity may be heightened. Wai et al. [51] observed that vector breeding was enhanced in the dry season in the Philippines due to the presence of water storage, conducive for mosquito growth and development. Tsuzuki et al. [52] also noted the potential of dengue transmission even in hot-dry periods in Nha Trang, Viet Nam, with similarly possible linkage to unchecked and left out indoor water containers.

Beyond the non-stationary relationship observed in the wavelet coherence patterns, we further investigated the impact of local meteorological variables on the dengue morbidity, with the primary focus on identifying the lag (weeks) in which dengue cases has occurred. Based on DLNM, there was a positive association between rainfall, average temperature and dew point with dengue cases. We found that, a moderate amount of rainfall has resulted with increasing RR and gradually decreased as the amount of rainfall increased.

Our findings are consistent with previous studies, whereby a high occurrence of dengue in the few weeks were also observed after moderate rainfall. Ehelepola et al. [53] have found that regular rain favors an increase in dengue but not heavy rain. Similarly, Sarfraz [54] noted that heavy rainfalls may flush away the eggs, larvae and pupae of dengue mosquitoes, which could have consequently affected the mosquito abundance [55]. On the other hand, we have observed that moderate amount of rainfall (20–30 mm) was related to higher dengue incidences in Davao Region. Eastin et al. [56] and Hii et al. [5] noted that light to moderate rainfall can increase the usage of water containers, which are conducive breeding sites for the mosquito.

According to DOH, Davao City which is located near Davao del Sur and a highly urbanized area in Davao Region, has always had the highest number of dengue, comprising around 70% of the cases [57] while the rest are from the neighboring provinces. Dengue is usually higher in highly urbanized areas like cities, where there is overcrowding and poor environmental waste management [58]. In 2013, there was a spike in dengue incidence in Davao City, which was assumed to be linked to the lack of cleanliness drive, unpredictable weather conditions and floods in the area [59] despite the increasing exertion of the DOH and the local health units in Davao Region on the implementation of dengue vector control programs throughout the years. One of the initiated clean-up activities by the DOH is the 4 o’clock habit, which entails the search and destruction of possible mosquito breeding sites, usually done in four in the afternoon [59].

Average temperature at 26 °C has resulted to an increased RR, while higher temperature from 27 °C to 31 °C has lower RR. This was suggestive of the established fact that mosquito development has an optimum range of 25 to 27 °C [60]. This optimum temperature for mosquito strongly enhanced the development from larva to adult, the biting frequency in humans, and the extrinsic incubation period of dengue virus in the mosquito. The decrease in the RR of temperatures from 27 °C to 31 °C is indicative that higher temperature above the optimum range for the *Aedes* mosquito development brings about a protective effect on dengue transmission [61], as observed in Fig. 5. Higher temperatures may have a negative effect on adult life span of mosquitoes, thereby affecting consequent transmission [62]. In particular, reduced vector competence and activity may result from an increased temperature [63].

Beyond a dew point temperature of 25.3 °C, risks were apparently increasing. Similar observations of significant relationship by dew point on dengue incidence was observed in Brazil [64]. Mechanisms of how dew point affects dengue incidence maybe related to that of the mechanisms posed by humidity. Mathematically and theoretically, dew point and humidity have a nearly linear relationship [65]. Taking this into account, we can observe an identical J-shaped pattern of absolute humidity and RR in a study done in Singapore [29] compared to Fig. 4. High humidity favors an increased longevity of adult mosquitoes as well as the shortening of viral incubation period, thereby allowing an increased transmission intensity [66].

We acknowledge that under-estimation of the burden poses one of the few challenges in establishing the robustness of the effects estimates in this study. However, even after applying an EF of 7, effects estimates as well as the risk curve remained the same, making the estimates robust (in Additional file 5: Figure S4). Furthermore, a recent study in the Philippines noted a high proportion of laboratory confirmed cases (86.1%) from the suspected cases, which thereby indicate that accuracy of clinical diagnosis at admission [67].

This is the very first study in the Philippines which extensively described the association between local meteorological variables and dengue incidences. This study could help improve the dengue surveillance system in the country by taking into account the underlying mechanisms which could be framed in the context of non-stationary relationship as well as the candidate threshold levels, for better dengue prediction. Furthermore, we also acknowledge some limitations in this study. First, the weekly dengue data used in this study were notified suspected dengue cases from clinics and hospitals and are not laboratory confirmed. Second, we did not take into account the mosquito density, population immunity, age classification, social behavior, and socioeconomic conditions for these data are unavailable.

## Conclusions

The meteorological variables, though may have varying effects on dengue incidence, exhibit associations which coincide with the plausible biological pathways. Risks were particularly higher in meteorological events with moderate rain, low temperature and high dew point. Furthermore, the observed possible threshold levels of these meteorological variables can be integrated into an early warning system to enhance dengue prediction for better vector control and management in the future.

## Additional files

Additional file 1: **Table S1.** *Sensitivity analysis with the combination of DLNM and simple linear models*. The table depicts that no matter the combination of the linear and DLNM models, there is not much improvement in the model performance, with similar observations in the previous univariate linear and DLNM models. (DOCX 14 kb)

Additional file 2: **Figure S2.** *Weekly distribution of dengue incidence, average temperature, and cumulative rainfall from 2011 to 2015 in Davao Region.* Red line is the cumulative rainfall, dotted blue line are the dengue incidences, and the green dot-and-line is the average temperature. The right-hand side y-axis is in degrees Celsius (for the average temperature), while the left-hand side y-axis is for the dengue incidence and rainfall levels. (JPEG 38 kb)

Additional file 3: **Figure S3.** *Distribution of monthly dengue incidences and local meteorological variables from 2011 to 2015*. (A) Box plot of the monthly dengue incidences, (B) Average temperature (°C), (C) Cumulative rainfall (mm), and (D) Dew point (°C). The horizontal line in the middle of each box is the mean, while the top and bottom borders of the box represent the 25th and 75th percentiles, respectively and the whiskers indicates the 10th and 90th percentiles. (TIF 234 kb)

Additional file 4: **Figure S1.** *Annual distribution of cumulative rainfall (mm) in Davao Region, 2011–2015.* Annual variations in the rainfall, with 2012 recording the highest level (of rainfall). (TIFF 6784 kb)

Additional file 5: **Figure S4.** *EF-applied dose-response risk curve***.** Relatively the same risk patterns can be observed, with heightened risks for moderate rain, low temperature and high dew point. (TIFF 1551 kb)

## Abbreviations

ASEAN: Association of South East Asian Nations
DHF: Dengue haemorrhagic fever
DLNM: Distributed lag nonlinear model
DOH: Department of Health
EF: Expansion Factor
NCDC: National Climatic Data Center
NCS: Natural cubic spline
NOAA: National Oceanic and Atmospheric Administration
PAGASA: Philippine Atmospheric Geophysical and Astronomical Services Administration
QAIC: Quasi-Akaike Information Criterion
RMSE: Root Mean Squared Error; RR – Relative risk
