# Supplementary material for: Meteorological factors affecting dengue incidence in Davao, Philippines
Source: BMC Public Health. 2018 May 15;18:629. doi: 10.1186/s12889-018-5532-4 (PMC5952851; doi:10.1186/s12889-018-5532-4)
Supplement: Supplementary file 1 — Table S1. Sensitivity analysis with the combination of DLNM and simple linear models. The table depicts that no matter the combination of the linear and DLNM models, there is not much improvement in the model performance, with similar observations in the previous univariate linear and DLNM models. (DOCX 14 kb) [file 12889_2018_5532_MOESM1_ESM.docx]

Table S1. Sensitivity analysis with the combination of DLNM and simple linear models

| **Models** |  | **QAIC** | **RMSE** | **pseudo R-squared** |
| --- | --- | --- | --- | --- |
| **Intercept only** |  | 13869.39 | - | - |
| **Simple Linear Models** |  |  |  |  |
|  | w/ Rainfall | 6088.93 | 0.3292689 | 0.6785798 |
|  | w/ Average temperature | 5866.015 | 0.3222838 | 0.6954379 |
|  | w/ Dew point | 5456.907 | 0.3183454 | 0.7251268^a^ |
|  |  |  |  |  |
| **DLNM Models** |  |  |  |  |
|  | w/ Rainfall | 4313.961 | 0.241543 | 0.8085139 |
|  | w/ Average temperature | 4291.979 | 0.251437 | 0.8112628 |
|  | w/ Dew point | 4002.741 | 0.2350322 | 0.8321903^b^ |
| **DLNM Models + Simple Linear models** |  |  |  |  |
|  | DLNM Precip + linear Tave + linear dewpt | 4060.791 | 0.2267171 | 0.8377105 |
|  | DLNM Tave + linear Precip + linear dewpt | 4188.607 | 0.2415359 | 0.8300417 |
|  | DLNM Dewpt + linear Tave + linear Precip | 3970.833 | 0.2268401 | 0.845221^c^ |

QAIC, Quasi-Akaike Information Criterion; RMSE, Root Mean Squared Error

^a^Best predictor among simple linear models

^b^Best predictor among the DLNM models

^c^Best predictor among the DLNM + Simple linear models

* Precip = precipitation; Tave = average temperature; dewpt = dew point
