# Supplementary figures and images for: Meteorological factors affecting dengue incidence in Davao, Philippines
Source: BMC Public Health. 2018 May 15;18:629. doi: 10.1186/s12889-018-5532-4 (PMC5952851; doi:10.1186/s12889-018-5532-4)

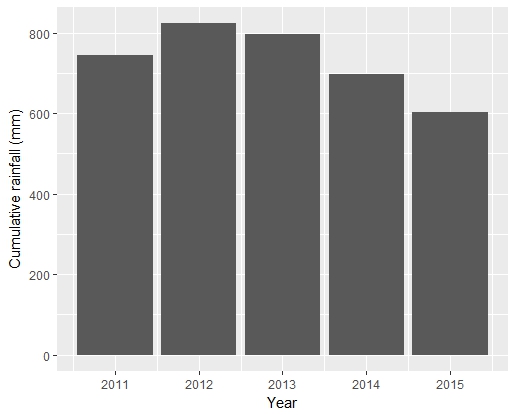

Supplement: Supplementary file 2 — Figure S2. Weekly distribution of dengue incidence, average temperature, and cumulative rainfall from 2011 to 2015 in Davao Region. Red line is the cumulative rainfall, dotted blue line are the dengue incidences, and the green dot-and-line is the average temperature. The right-hand side y-axis is in degrees Celsius (for the average temperature), while the left-hand side y-axis is for the dengue incidence and rainfall levels. (JPEG 38 kb) [file 12889_2018_5532_MOESM2_ESM.jpeg]

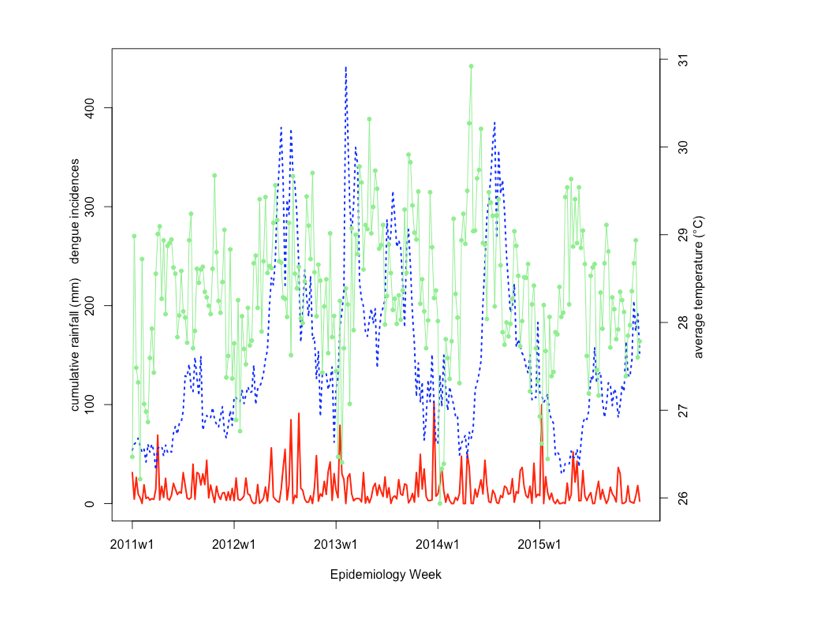

Supplement: Supplementary file 3 — Figure S3. Distribution of monthly dengue incidences and local meteorological variables from 2011 to 2015. (A) Box plot of the monthly dengue incidences, (B) Average temperature (°C), (C) Cumulative rainfall (mm), and (D) Dew point (°C). The horizontal line in the middle of each box is the mean, while the top and bottom borders of the box represent the 25th and 75th percentiles, respectively and the whiskers indicates the 10th and 90th percentiles. (TIF 234 kb) [file 12889_2018_5532_MOESM3_ESM.tif]
